# 462. Infant antibody titers at birth following maternal COVID-19 vaccination and protection against infection in the first 6 months of life

**DOI:** 10.1093/ofid/ofad500.532

**Published:** 2023-11-27

**Authors:** Cristina Cardemil, Flor M Munoz, Yi Cao, Fei Gao, Christine M Posavad, Martina L Badell, Katherine E Bunge, Mark J Mulligan, Lalitha Parameswaran, Courtney Olson-Chen, Richard M Novak, Rebecca C Brady, Emily A DeFranco, Jeffrey S Gerber, Marcela Pasetti, Mallory C Shriver, Rhea Coler, Bryan J Berube, Barbra A Richardson, Richard H Beigi, Kathy M Neuzil, Elizabeth R Brown

**Affiliations:** National Institutes of Health, Rockville, Maryland; Baylor College of Medicine, Houston, TX; Fred Hutchinson Cancer Center, Seattle, Washington; Fred Hutchinson Cancer Center, Seattle, Washington; Fred Hutchinson Cancer Center, Seattle, Washington; Emory University School of Medicine, Atlanta, Georgia; University of Pittsburgh, Pittsburgh, Pennsylvania; NYU Grossman School of Medicine, New York, New York; NYU Langone Health, NYU Langone Vaccine Center, New York, New York; University of Rochester, Rochester, New York; University of illinois at Chicago, Chicago, Illinois; CIncinnati Children's Hospital Medical Center, Cincinnati, Ohio; Cincinnati Children’s Hospital Medical Center, Division of Infectious Diseases, University of Cincinnati College of Medicine, Cincinnati, Ohio; Children's Hospital of Philadelphia, Philadelphia, Pennsylvania; University of Maryland, Baltimore, Maryland; University of Maryland School of Medicine, Baltimore, Maryland; Seattle Children’s Research Institute, Center for Global Infectious Disease Research, Seattle, Washington; Seattle Children's Research Institute, Seattle, Washington; University of Washington, Seattle, Washington; UPMC Magee-Womens Hospital, Pittsburgh, Pennsylvania; University of Maryland School of Medicine, Baltimore, Maryland; Fred Hutchinson Cancer Center, Seattle, Washington

## Abstract

**Background:**

Maternal vaccination is a promising strategy to prevent COVID-19 in early infancy, yet the amount of protection that maternally derived vaccine specific SARS-CoV-2 binding and neutralizing antibodies provide to infants in the first six months of life is not well characterized.

**Methods:**

Infants born to mothers vaccinated during pregnancy with 2 or 3 doses of an mRNA COVID-19 vaccine (primary series or boosted group, respectively) were followed prospectively from birth up to 6 months. At delivery, anti-Spike IgG and pseudovirus neutralizing antibody (Nab) levels were measured. Between September 2021 and December 2022, at pre-specified timepoints, COVID-19 infection was determined by verified maternal report. The risk reduction for COVID-19 in infants by antibody titers at delivery was estimated using a calendar-time Cox regression model after adjusting for maternal age, booster status, and recency of last dose. The relative vaccine effectiveness (VE) of 3 vs 2 maternal vaccine doses for COVID-19 in infants < 6 months was estimated in separate models.

**Results:**

Infants in the boosted group (n=181) had higher anti-Spike IgG and pseudovirus Nab titers at delivery compared with the primary series group (n=260) (mean 4443 vs 1091 BAU/ml and 1567 vs 404 IC50, respectively; both p< 0.001). A 10-fold increase in anti-Spike IgG measured at delivery was associated with a 69.2% (95% CI: 38.1%, 84.6%, p=0.001) reduction in the infant’s risk of acquiring COVID-19 in the first 6 months. Similarly, having a pseudovirus Nab response above an IC50 of 20 at delivery was associated with a 96.2% (95% CI: 75.1%, 99.4%; p=0.001) reduction in the infant’s risk of acquiring COVID-19 in the first 6 months. The relative VE of 3 vs 2 doses against infant COVID-19 was 65% (95% CI: 24%, 84%) when accounting for changes in anti-Spike IgG levels, and 83% (95% CI: 43%, 95%) when accounting for pseudovirus Nab levels.

**Conclusion:**

Higher SARS-CoV-2 IgG Spike and pseudovirus Nab titers at delivery are associated with a substantially reduced risk of COVID-19 infection for infants in the first 6 months of life. Until infants are age-eligible for COVID-19 vaccination, maternal vaccination provides transplacentally transferred passive binding and neutralizing SARS-CoV-2 antibodies that protect against infection during early infancy.

**Disclosures:**

**Flor M. Munoz, MD, MSc**, CDC respiratory virus surveillance: Grant/Research Support|Gilead: Grant/Research Support|Moderna, sanofi, aztra zeneca, Merck, GSK: Advisor/Consultant|NIH: DSMB|NIH COVID-19 vaccines in pregnancy: Grant/Research Support|Pfizer Pediatric COVID-19 vaccines: Grant/Research Support|Pfizer, Dynavax, Monderna, Meissa, NIH: DSMB **Mark J. Mulligan, M.D.**, Lilly: Grant/Research Support|Meissa Vaccines, Inc.: Advisor/Consultant|Meissa Vaccines, Inc.: Board Member|Merck: Advisor/Consultant|Merck: Board Member|Pfizer: Advisor/Consultant|Pfizer: Board Member|Pfizer: Grant/Research Support|Sanofi: Grant/Research Support **Lalitha Parameswaran, MD, MPH**, Pfizer: Grant/Research Support **Richard M. Novak, MD**, Moderna: Advisor/Consultant **Rebecca C. Brady, MD**, AztraZeneca: Grant/Research Support|PATH: Grant/Research Support|Pfizer: Grant/Research Support **Bryan J. Berube, PhD**, HDT Bio Corp.: Author on patents|HDT Bio Corp.: Salary|HDT Bio Corp.: Ownership Interest **Barbra A. Richardson, PhD**, Gilead Sciences: Advisor/Consultant **Kathy M. Neuzil, MD, MPH**, NIH: Grant/Research Support|Pfizer: Grant/Research Support **Elizabeth R. Brown, ScD**, Merck: Advisor/Consultant

